# Eggshell waste as a bioremoval agent for potentially toxic elements/metals and microbial contaminants from raw water of the Nile River in Egypt

**DOI:** 10.1186/s13104-025-07199-y

**Published:** 2025-04-15

**Authors:** Alaa Hosny, Nada Wael, Menna A. Hossam, Mariam Abdelmonem, Salwa M. El-Sayed, Samah H. Abu-Hussien, Basma T. Abd-Elhalim

**Affiliations:** 1 New Programs, Faculty of Agriculture, Ain Shams University, PO Box 68-Hadayek Shoubra, Cairo, 11241 Egypt; 2 Department of Biochemistry, Faculty of Agriculture, Ain Shams University, PO Box 68-Hadayek Shoubra, Cairo, 11241 Egypt; 3 Department of Agricultural Microbiology, Faculty of Agriculture, Ain Shams University, PO Box 68-Hadayek Shoubra, Shubra El-Khaimah, Cairo, 11241 Egypt

**Keywords:** Bioremediation, Eggshell waste, Potentially toxic elements/metals, Microbial contamination, Nile river, Raw water

## Abstract

Recently, microbial, and potentially toxic elements/metals (PTEs) contamination of aquatic ecosystems has been increasing in Egypt, owing to the bio-disposal of such pollutants in water effluents. This study focused on using Eggshell waste (ESW) as a bioremoval agent for metals and microbial contaminants from raw water of the Nile river in Egypt which considered the source for life for all Egyptians. ESW was collected from local bakeries in Cairo, Egypt, and prepared for use as bioadsorbent. All raw water samples were treated with prepared ESW and tested for initial and end concentrations of PTEs and microbial load contents. Moreover, Scanning Electron Microscopy with Energy Dispersive X-ray analysis (SEM-EDX) was performed to test ESW characterization properties before and after raw water treatment using ESW. Results obtained by SEM recorded irregular rhombus-like stereo structures with tiny pore structures and lamellar structures with enlarged pore architectures dispersed randomly on the surface before ESW treatment. After ESW treatment, SEM-EDX results indicated a regular and adhesive appearance on the surface of ESW. Moreover, current results revealed that bioremoval efficiency reached 94.4, 64.7, and 51.4% for removing lead, cadmium, and iron, using ESW, respectively. Moreover, ESW was highly effective in eliminating *Escherichia coli* throughout the first 4 h of contacting and inhibiting 70% of the microbial load incubated at 37 °C, and complete inhibition occurred after 24 h of contacting process. Overall, this study advances knowledge in bioremediation and offers practical solutions for water quality management using organic waste materials.

## Highlights

This study investigates the potential of eggshell waste (ESW) as a sustainable bioremoval agent for effectively removing potentially toxic elements/metals (PTEs) and microbial contaminants from raw water of the Nile River in Egypt.

The findings demonstrate that eggshell waste achieved removal efficiencies of 94.4% for lead, 64.7% for cadmium, and 51.4% for iron, showcasing its effectiveness in adsorbing harmful metals from water sources.

ESW proved highly effective in eliminating *Escherichia coli*, achieving complete inhibition after 24 h of contact, and showing significant reduction in bacterial counts within the first 4 h.

Scanning Electron Microscopy with Energy Dispersive X-ray analysis (SEM-EDX) revealed structural changes in ESW before and after treatment, indicating its significant adsorption capabilities.

The study highlights the feasibility of utilizing agricultural by-products like ESW as a green alternative for water purification, promoting both waste reduction and environmental sustainability.

Given the context of increasing water pollution in Egypt, this research provides insights that could inform local practices for improving water quality and protecting public health.

These highlights encapsulate the key findings and significance of the study, emphasizing its contributions to the field of bioremediation and sustainable water management.

## Introduction

Water pollution stands as a critical global issue, posing serious threats to human health and ecosystems alike [1]. In Egypt, the Nile River—a vital water source for millions—is increasingly subjected to contamination from various pollutants, including potentially toxic elements (PTEs) and pathogenic microorganisms. The presence of these contaminants can lead to significant health risks, contributing to chronic conditions such as neurological disorders and organ damage due to heavy metal exposure, along with outbreaks of waterborne diseases associated with microbial contamination [2].

Addressing water pollution necessitates the exploration of innovative and sustainable treatment solutions. Conventional water treatment methods often rely on costly chemical processes and advanced technologies that may not be accessible or practical in many regions, particularly in developing countries [3]. As a result, the search for eco-friendly and economically viable alternatives has become essential [2, 4]. Bioremediation has emerged as a promising strategy for tackling water pollution, leveraging biological materials to mitigate or eliminate contaminants while promoting environmental sustainability [5].

In this context, eggshell waste (ESW) represents a unique and underutilized resource. Commonly produced as a by-product of agriculture, eggshells are predominantly composed of calcium carbonate, which possesses innate properties that can facilitate the adsorption of heavy metals and microbial contaminants [6, 7]. Utilizing ESW not only aids waste management but also aligns sustainable practices by reducing the environmental impact associated with waste disposal [8, 9].

This study investigates the potential of eggshell waste as a bioremoval agent for PTEs and microbial contaminants from raw water sourced from the Nile River. Through the characterization of ESW and an assessment of its adsorption capabilities, this research aims to demonstrate the effectiveness of this agricultural resource in enhancing water quality. Ultimately, the findings intend to contribute to public health safety and the promotion of sustainable practices in water resource management in Egypt.

## Materials and methods

### Eggshell waste (ESW) collection and preparation

Egg shell waste (ESW) samples were collected in sterilized plastic bags from local bakery markets in Cairo, Egypt. ESW samples were transported to the laboratory and kept at 4 ℃ for further studies. For preparing eggshell waste powder (EWP), ESW was rinsed several times to remove impurities and interference substances like salts and organic acids. Thereafter, all ESW was boiled at 100 ℃ for 15 min and dried at 150 ℃ for 30 min in a hot-air sterilizer. All dried samples were crushed using an electric grinder (Moulnix hand blinder, France) then the powder was kept in a dark place for further investigations [10].

### Raw water samples collection

**Fig. 1:**
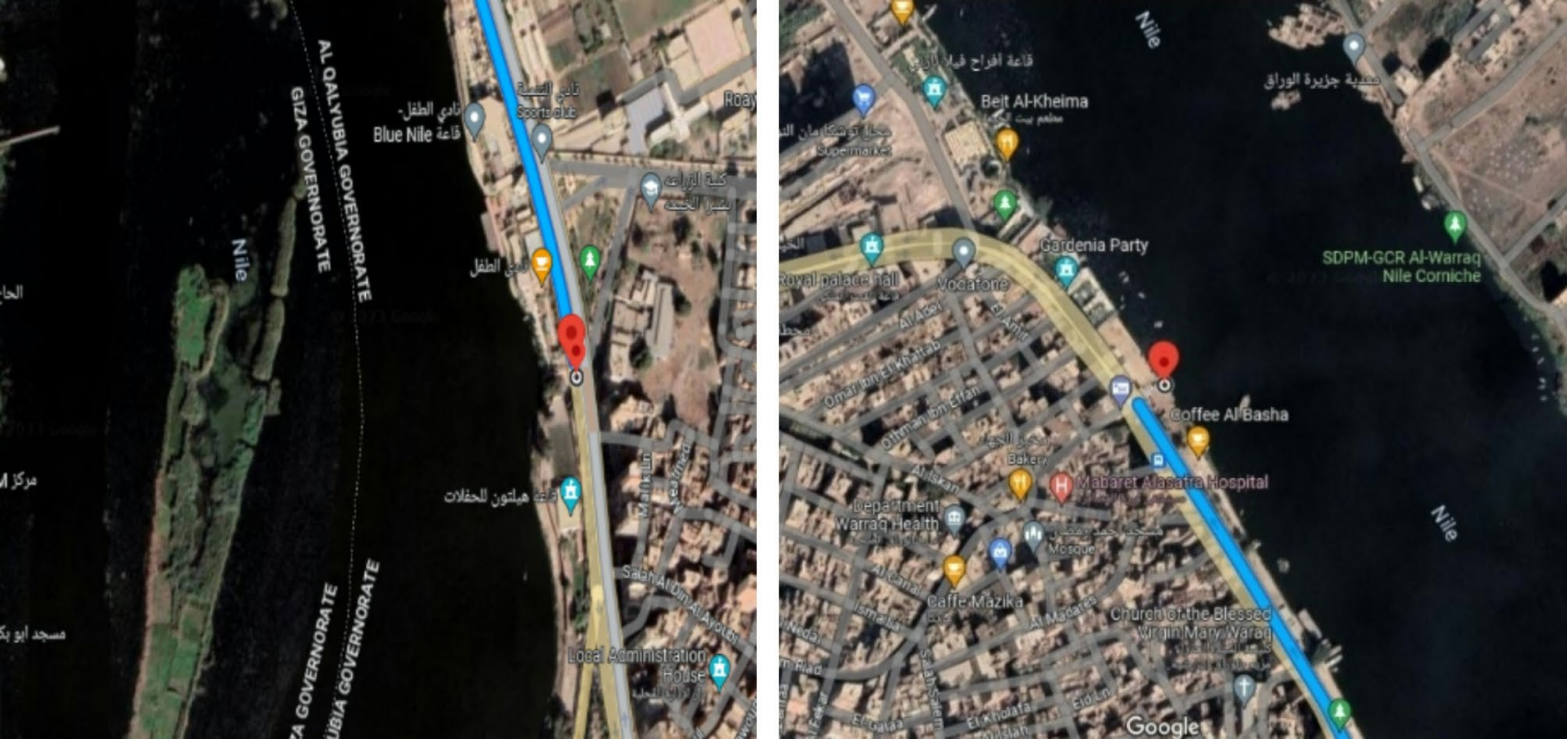
Locations of raw water samples collection at Shubra El-Khaima, Cairo, Egypt

Raw water samples were collected from three different places at Nile River, Shubra El-Khaima, Cairo, Egypt (Fig. 1). The samples location was in 30°06’19.8”N 31°13’13.9”E, and 30°06’38.7”N 31°14’36.3”E.

### Raw water treatment with ESW

Briefly, 30.0 g of ESW was added to 100 mL of raw water sample in a 500 mL Erlenmeyer flask and incubated at 37 °C for 24 h. Samples were taken at 2 h intervals. Ten mL of collected samples were taken for ESW to assess its PTEs and microbial load contents before and after raw water treatment.

### Characteristics of ESW using SEM-EDX analysis

The image of the surface of ESW was obtained by SEM-EDX (Tescan Vega 3 SBU, Czech Republic) at Medical and Scientific Centre of Excellent, Giza, Egypt, to study the morphological characteristics of ESW before and after raw water treatment as a biosorbents. Also provides a rapid non-destructive estimation of PTEs composition in ESW samples. Samples were mounted on aluminum microscopy tubs using carbon tape, then coated with gold (Au) for 100 s using Quorum techniques Ltd, sputter coater (Q150t, England), and Magnified (10.00 kx, 20 μm). EDX is an X-ray technique used to identify the elemental composition of materials. The data generated by EDX analysis consists of spectra showing peaks corresponding to the elements, indicating the true composition of ESW sample [11].

### Potentially toxic elements/metals (PTEs) analysis by inductively coupled plasma mass spectrometry (ICP-MS)

Potentially toxic elements/metals concentration was determined in raw water samples before and after adding ESW as the guidelines of [12, 13]. ICP-MS technique was used to identify all potentially toxic elements/metals based on the ionization of the elements within the sample as illustrated by [14, 15].

### Total bacterial count and *E. coli* detection

All treated raw water samples were collected as previously described at 2 h intervals for mounting total bacterial count using plate count assay [16]. Briefly, all collected samples were ten-folds serially diluted and then 1 mL of each dilution was cultured on both nutrient agar and Mackonkey agar media provided from Oxoid, India, incubated at 37 °C for 24 h. *E. coli* colonies were observed, and total microbial load was counted, and the results were expressed as colony forming unit per milliliter (CFU/mL).

### Statistical analysis

Statistical differences among data from compressive strength results were determined using analysis of variance (ANOVA) with Statistics^®^ software. Duncan’s significant difference test was conducted after the determination of variances (*p* ≥ 0.05) [17].

## Results

### Physiochemical characterization of ESW using SEM-EDX before raw water treatment

**Fig. 2:**
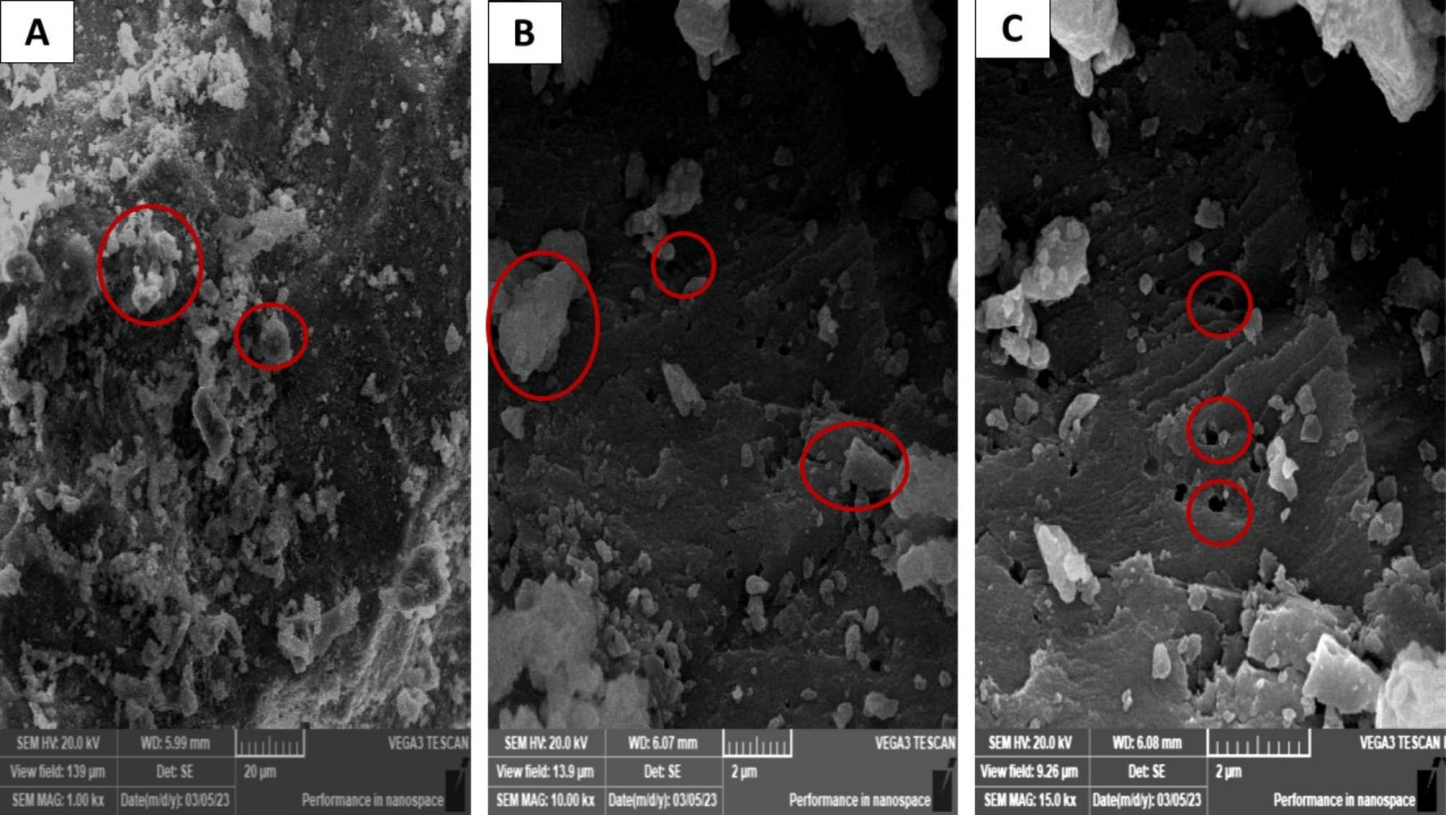
Scanning Electron Microscopy (SEM) images of eggshell waste (ESW), **A)** before treatment with raw water, **B**) Red circles show irregular rhombus-like stereo structure with tiny pore structures, **C**) Red circles show a lamellar structure with enlarged pore architectures dispersed randomly on the surface at 20 μm of magnification scale

The ESW was assessed for its structural, textural, and other characteristics using SEM-EDX tool before and after raw water treatment. ESW after drying at 150 °C had a lamellar structure with enlarged pore architectures, while conventional drying of ESW before treatment showed an irregular rhombus-like stereo structure with tiny pore structures dispersed randomly on the surface at 20 μm of magnification scale, Fig. 2.

**Table 1:** Energy dispersive X-ray analysis (EDX) characterization of egg shell waste (ESW) before water treatment and potentially toxic elements/metals (PTEs) absorbance

Element	Atomic number	Mass (%)	Mass normal (%)	Atom (%)
Oxygen	8	29.71	49.4	63.29
Carbon	6	4.180	6.95	11.86
Nitrogen	7	1.570	2.61	3.81
Calcium	20	24.49	40.8	20.83
Magnesium	12	0.14	0.24	0.20
Sulfur	16	0.01	0.01	0.01
Potassium	19	0.08	0.00	0.00
Iron	26	0.16	0.00	0.00
		60.09	100.00	100.00

**Fig. 3:**
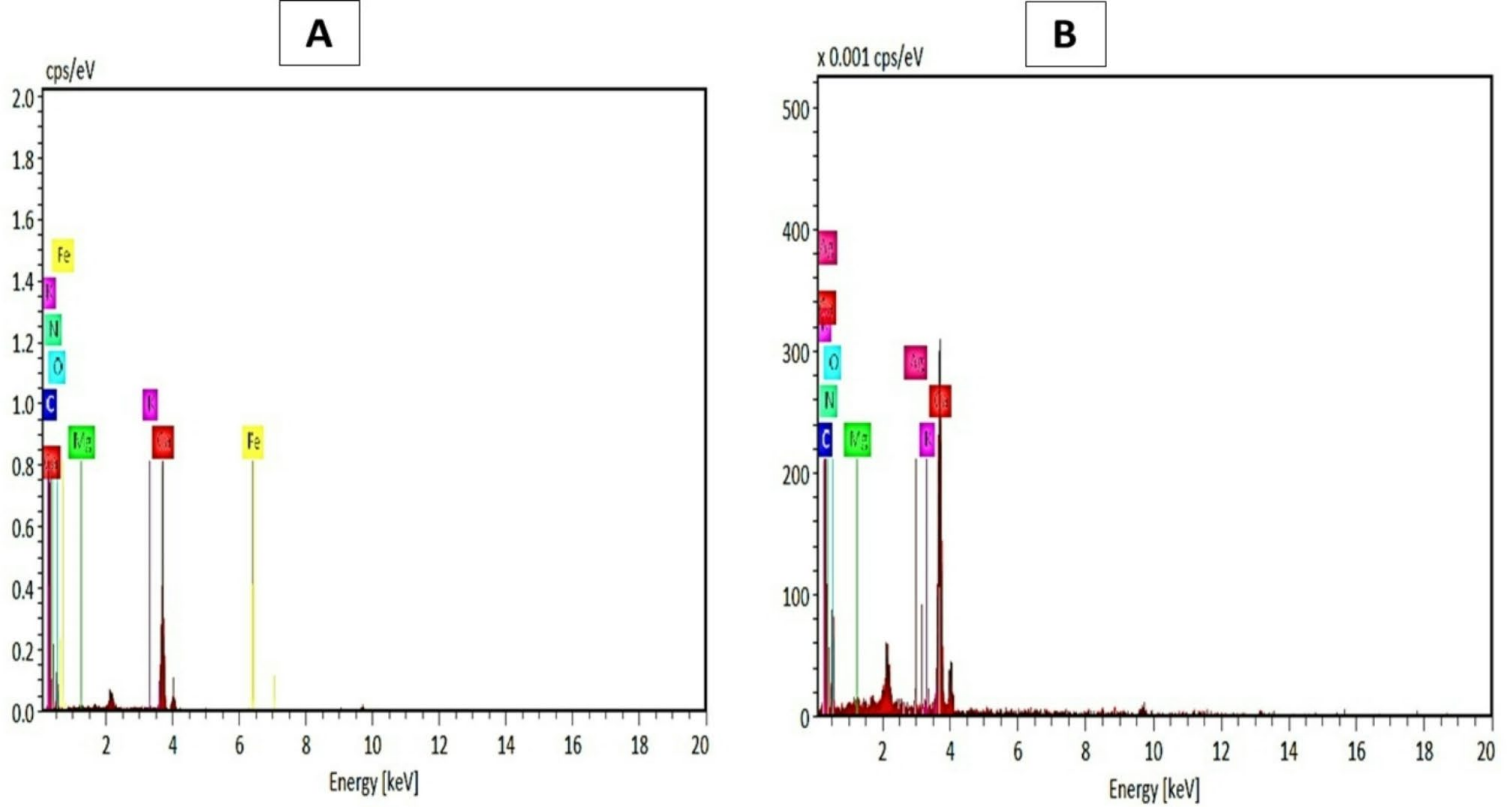
Energy Dispersive X-Ray analysis (EDX) of Egg shell powder (ESW) **A**) before potentially toxic elements/metals absorbance. **B**) Heave metals presence in the eggshell at two different scanning fields

The EDX analysis in Fig. 3; Table 1 illustrated the percentage of (%) heavy metals and their oxides that are attached to eggshell (ES-PTEs complex). Oxygen, carbon and nitrogen masses were significantly higher than all other elements, as they are the major components of eggshells. Mass percentages for metals were 29.71% for Oxygen, 4.18% for Carbon, 1.57% for Nitrogen, 24.49% for Calcium, 0.14% for Magnesium, 0.01% for Sulphur, 0.08% for Potassium and 0.16% for Iron.

### Physiochemical characterization of ESW after raw water treatment

**Fig. 4:**
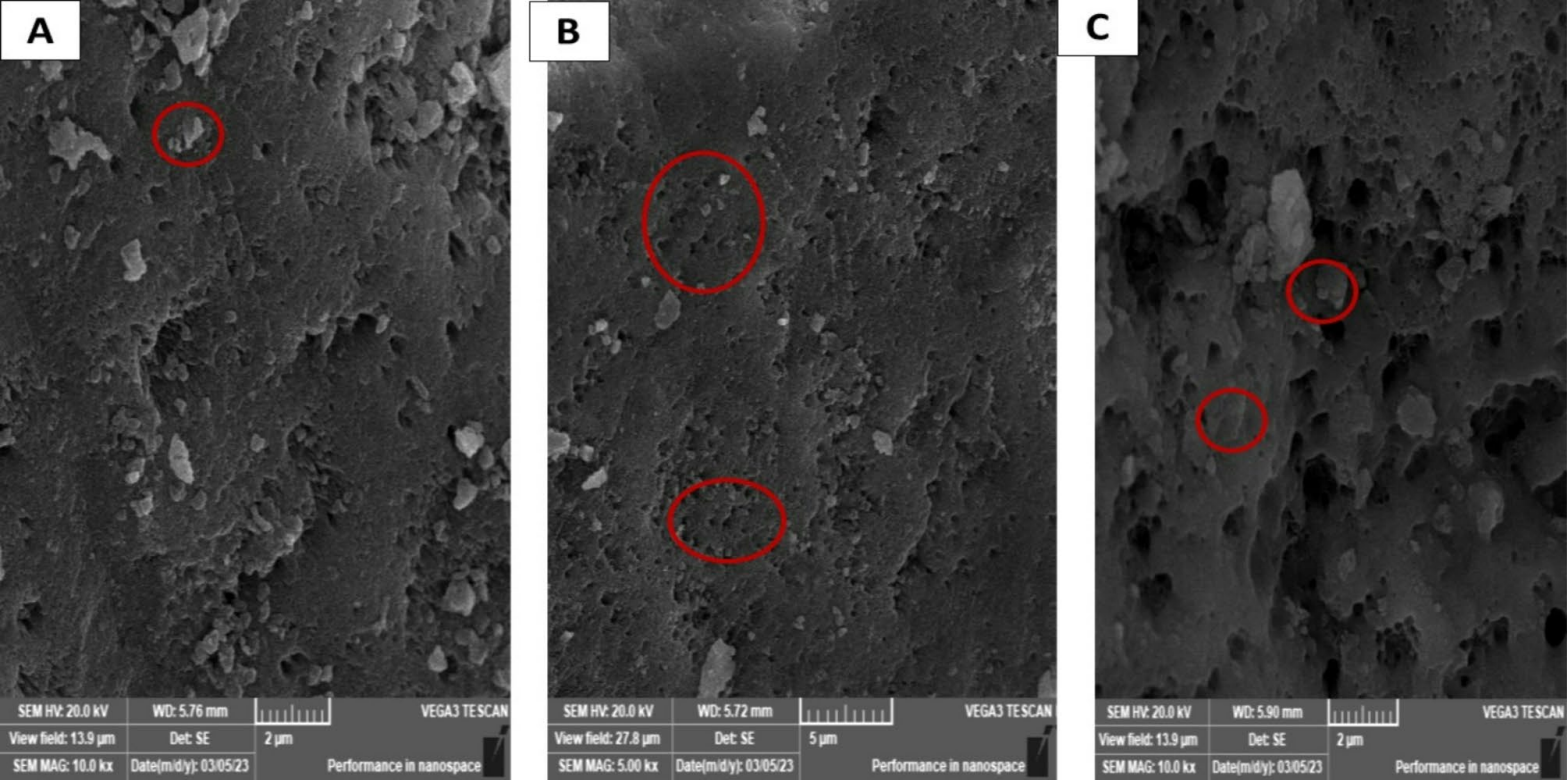
Scanning Electron Microscopy (SEM) images of eggshell waste (ESW) after treatment with raw water. Red circles show regular and adhesive appearance on the surface of ESW at 20 μm of magnification scale

**Fig. 5:**
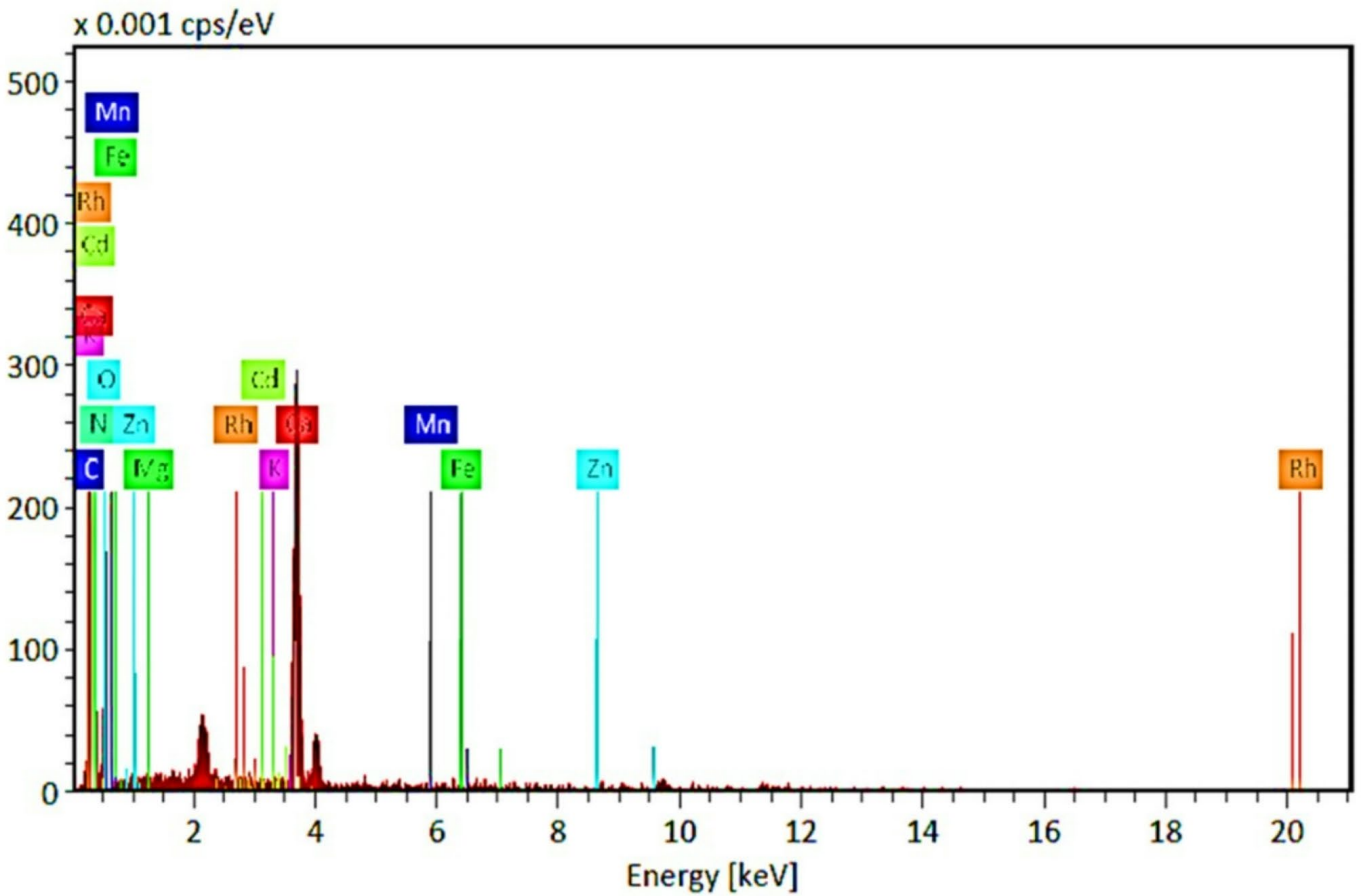
Energy dispersive X-ray analysis (EDX) of Egg shell waste (ESW) after potentially toxic elements/metals absorbance

Morphological studies of eggshell surface with adsorbed metals after treatment with raw water was studied using SEM technique as shown in Fig. 4A-C. SEM analysis pointed to destruction happened in ES matrix structure at magnification scale of 20 μm. After potentially toxic elements/metals adsorption, ES showed regular and adhesive appearance. Figure 5; Table 2 show mass percentages for the studied metals. No significant differences were recorded for basic metal masses (Oxygen, Carbon, Nitrogen) percentage before and after raw water treatments, while there is a high existence for various potentially toxic elements/metals after treating raw water with ESW.

**Table 2:** Energy dispersive X-ray analysis (EDX) characterization of egg shell waste (ESW) after Raw water treatment and potentially toxic elements/metals absorbance

Element	Atomic number	Mass (%)	Mass normal (%)	Atom (%)
Oxygen	8	23.6	40.1	52.7
Carbon	6	5.26	8.94	15.6
Nitrogen	7	3.07	5.22	7.00
Calcium	20	25.7	41.0	22.9
Zinc	30	0.46	0.77	0.25
Cobalt	27	0.17	0.26	0.10
Copper	29	0.24	1.16	0.42
Magnesium	12	0.26	0.40	0.39
Aluminum	13	0.18	0.27	0.23
Manganese	25	0.06	0.10	0.04
Niobium	41	0.18	0.27	0.06
Lead	82	0.23	0.37	0.04
Iron	26	0.13	0.09	0.03
Rhodium	45	0.05	0.08	0.02
Potassium	19	0.04	0.06	0.03
Nickel	28	0.23	0.34	0.12
Cadmium	48	0.34	0.57	0.11
		60.2	100.00	100.0

### Microbial and PTEs removal efficiency of ESW

Table 3 showed the removal efficiency of raw water microflora and its PTEs composition. Results recorded a significant increase of Lead, Cadmium and Iron percentage after treatment reaching removal efficiency of 94.4%, 64.7% and 51.4% respectively. Moreover, ESW had a high efficiency for the removal of microbial contaminants from treated raw water. Table 3 also illustrated the difference between microbial counts before and after raw water by the ESW.

**Table 3:** Removal efficiency of Raw water microflora and its potentially toxic elements/metals composition using ESW before and after Raw water treatment

**PTEs removal efficiency**	**Metal Ion**	**Before ESW treatment**	**After ESW treatment**
	Lead (Pb)	0.45^e^ ± 0.02	0.0250^a^ ± 0.44
	Cadmium (Cd)	0.88^f^ ± 0.11	0.3103^d^ ± 0.01
	Iron (Fe)	0.25^c^ ± 0.14	0.1214^b^ ± 0.21
**Microbial removal efficiency**	**Incubation time (h)**	**Total bacterial count (CFU/mL)**	***E. coli*** **detection**
	(before treatment)	128	+ +
	0	128	+
	2	128	*-*
	4	51	*-*
	6	30<	*-*
	24	30<	*-*

*(++) = strong, (+) = moderate, and (-) = no growth

## Discussion

Inorganic contaminants in water sources, particularly potentially toxic elements/metals, originate mostly from many sources such as pesticides, metal finishing, fertilizers, wastewater, power plants, animal manure, battery manufacture, and sewage sludge [18]. Heavy metals, unlike organic contaminants, are non-biodegradable, which makes them have a high toxicity to all the living organisms [19]. So that the removal efficiency of the ESW was a major perspective. The EDX analysis illustrated the percentage of potentially toxic elements/metals and their oxides that are attached to eggshell. Oxygen, carbon and nitrogen masses were significantly higher than all other elements, as they are the major components of eggshells. Mass percentages for metals were 29.71% for O, 4.18% for C, 1.57% for N, 24.49% for Ca, 0.14% for Mg, 0.01% for S, 0.08% for K and 0.16% for Fe.

As shown the ESW has a great potency to remove potentially toxic elements/metals of Al, Fe, and Zn from water. As it showed a high affinity to Al, more than the Fe and Zn, as the removal percentage was from 30 to 97%. Also, ESW was reported by [20, 21] on bioremediation of potentially toxic elements/metals of mine drainage, and their observations revealed that 100% removal of Al and Fe and partial removal of Mn occurred. In addition [15], examined the calcined eggshells were used to get the red of the PTEs from acid mine drainage, and the percent of Mn, Zn, and Fe were 80.5, 80.2 and 80.3%, respectively.

Morphological studies of ESW surface with adsorbed metals after treatment with raw water was studied using SEM technique and it was pointed that the destruction happened in ESW matrix structure at magnification scale of 20 μm. After potentially toxic elements/metals adsorption, ESW showed regular and adhesive appearance with no significant differences were recorded for basic metal masses (O, C, N) % before and after raw water treatments, while there is a high existence for various potentially toxic elements/metals after treating raw water with ESW. The percentage of masses were 23.6% for O, 5.26% for C, 3.07% for N, 25.7% for Ca, 0.26% for Mg, 0.04% for K, 0.24% for Cu, 0.23% for Pb, 0.18% for Al, 0.17% for Co, 0.23% for Ni, 0.06% for Mg,0.46% for Zn, 0.05% for Fe, 0.18% for Nb, and Rh 0.05% as discussed by [5, 22–24].

As the ESW will dissolve partially and release Ca^+ 2^, CO_3_^-2^, HCO_3_^-^ and OH^-^ ions and this can contribute to enhancing the uptake of metal ions mechanism which is as follows; follows the cationic exchange properties of metal and the calcium ion exchange process then the electrostatic interaction between the negative charge species (e.g., carbonate ion) and the positive charge of metal ions. In the presence of water, CaCO_3_ would undergo displacement reaction as reaction equation:

1 $$\:{CaCO}_{3}\hspace{0.17em}+\hspace{0.17em}{H}_{2}O\iff\:\:{Ca}^{+\hspace{0.17em}2}\:+\:{HCO}_{3}-\:+\:{OH}^{-}\:+\:{CO}_{2}^{-3}$$

**Fig. 6:**
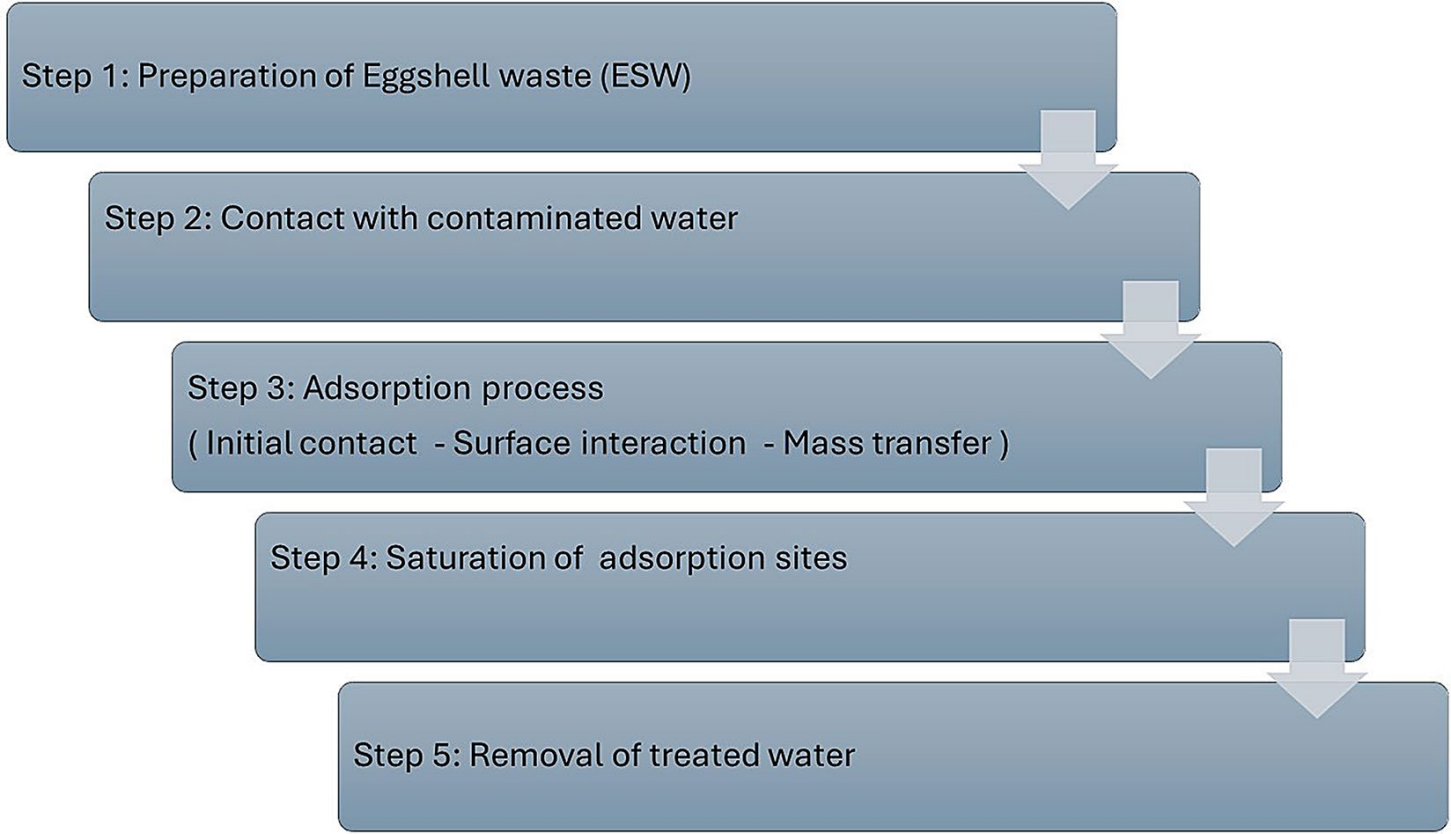
Step-by-step flowchart explanation of the ESW adsorption mechanism

The adsorption process of metal pollutants using ESW involves several key steps. Here is a step-by-step explanation of the adsorption mechanism (Fig. 6):

1. Initial contact: Metal ions begin to interact with the surface of the ESW as soon as they come into contact.
2. Surface Interaction.
3. Electrostatic attraction: Positive charges on the metal ions attract anionic sites (like oxygen and carbonyl groups) on the eggshell surface.
4. Physical adsorption: Weak Van der Waals forces play a role in stabilizing the metal ions on the surface.
5. Chemical adsorption: Coordination bonds may form between the metal ions and functional groups (such as -COOH and -OH) present on the surface of the ESW.
6. Diffusion: Metal ions diffuse from the bulk solution to the surface of the biosorbent. This step can be influenced by concentration, gradients and temperature.
7. Filtration: The larger metal ions get filtered into the porous structure of the ESW, allowing for retention.
8. Completion of Adsorption: As the adsorption sites on the eggshells become occupied, the capacity for further adsorption decreases.
9. Equilibrium: The process reaches equilibrium when there is no significant change in the concentration of metal ions in the solution.
10. Separation: Once the adsorption is complete, the water treated is separated from the ESW through filtration or sedimentation.

The following pictorial flowchart of the adsorption mechanism visually summarizes the adsorption mechanism of target metal pollutants by ESW:

With this flowchart, we can see the consecutive steps that illustrate the effective adsorption of metal pollutants by ESW. Each step contributes to the overall efficacy of the bioremediation process, demonstrating the potential of ESW as a natural and sustainable adsorbent for environmental cleanup efforts.

In the same line with our findings [5, 25–27], mentioned the efficiency of EWS as potentially toxic elements/metals adsorbent as exhibited maximum values of 32.57 mg/g for Zn, 30.12 mg/g Cu, 2.56 mg/g Co, and 0.28 mg/g Ni. Results recorded a significant increase of Pb, Cd and Fe % after treatment reaching removal efficiency of 94.4%, 64.7% and 51.4% respectively. Moreover, ESW had a high efficiency for the removal of microbial contaminants from treated raw water.

As illustrated the difference between microbial counts before and after raw water by the ESW. After raw water treatments, samples were counted at 2 h intervals for 24 h to calculate the final ESW microbial load. After 2 h of treating with ESW, a significant reduction in microbial load was observed by efficiency of 60.2%. In addition, after 4 h of raw water treatment with ESW, no microbial visible growth was recorded, indicating a disinfectant effect of ESW. Moreover, *E. coli* was completely eliminated with removal percentage reached 99.99% after only 4 h of treatment. The microbial elimination using ESW was due to the nature created the eggshell to protect the embryo from dangerous microorganisms and other causes as the eggshell has multiple po res that trap pathogen elimination in this technique can largely happen through physical squeezing into tiny pore gaps and attachment [24, 28, 29]. The results of [13] revealed that there was a significant reduction in *E. coli* (99%) after 3 h of calcined eggshell contact. Then, after 6 h of contact, there was a complete removal of *E. coli*. In coincidence [25, 30], reported that eggshells led to the complete disinfection of *E. coli*.

### The desorption, reuse, and final disposal of ESW as an adsorbent

Incorporating ESW as a bioremoval agent for PTEs and microbial contaminants raises several important considerations regarding the post-treatment management of the adsorbent. Specifically, the stages of desorption, reuse, and final disposal of ESW can significantly impact the sustainability and overall effectiveness of using this organic material for water treatment.

1. The potential for desorption of contaminants from the ESW back into the water column must be assessed to ensure that treated water remains safe for use. Desorption can occur due to changes in environmental conditions such as pH, ionic strength, or competitive adsorption by other ions present in the water. Also, Laboratory studies exploring desorption mechanisms can help identify optimal conditions for effective contaminant removal without re-release. Investigating treatments such as pH adjustment or competitive ion displacement may reveal potential methods for minimizing desorption [21].
2. The possibility of regenerating and reusing ESW as an adsorbent after pollutant treatment is critical for enhancing the sustainability of this approach. Various regeneration techniques, such as heating, chemical leaching, or washing, can be evaluated to recover the adsorbent’s capacity for future use. It is essential to monitor the physicochemical properties of ESW post-regeneration to ensure that its adsorptive qualities remain intact. However, excessive regeneration processes may lead to structural degradation of the eggshell, thereby reducing its efficiency [23].
3. Implementing a reuse strategy must also consider the economic viability and lifecycle assessment of ESW as an adsorbent. Comparison with other commercially available adsorbents in terms of cost, efficiency, and operational feasibility should be conducted. The financial implications of regeneration versus the cost of disposal when ESW can no longer be effectively reused should guide decision-making regarding its application in bioremediation.
4. Once ESW has reached the end of its usable life or regeneration is no longer feasible, the final disposal approach must be environmentally sound. Options include composting, incorporation into soil amendments, or incineration, depending on regulatory frameworks and environmental considerations. Proper disposal methods can help ensure that any trapped contaminants are managed correctly and do not leach back into the ecosystem, potentially causing further contamination. For example, composting could enhance soil properties while safely immobilizing contaminants [28].
5. Final disposal strategies should adhere to local and international regulations governing waste management to ensure compliance and minimize environmental risks. Developments in policy can support sustainable practices for waste disposal, promoting the responsible use of organic materials like eggshell waste in various applications.

The study’s findings on utilizing ESW as a bioremoval agent provide valuable insights into the management of contaminated water sources; however, they also come with certain implications and limitations regarding the organic content and the overall effectiveness of the process. Here’s a discussion incorporating both aspects:

### Implications

1. Organic content interference:

- Adsorption efficiency: The organic matter present in raw water—such as humic substances, microorganisms, and other organic pollutants—can interfere with the adsorption capacity of the adsorbent. High levels of organic content may compete with toxic metals and microbial contaminants for available binding sites on the eggshells, potentially reducing the overall efficiency of the bioremoval process.

2. Nutrient release and microbial growth:

- Release of nutrients: The organic materials present can leach nutrients upon treatment. While this can promote microbial growth, which is beneficial for certain bioremediation processes, it could also facilitate the growth of pathogenic bacteria if not adequately managed.

3. Decomposition of organic content:

- Treatment Stability: Organic content decomposition can lead to changes in pH and chemical composition of the treated water, which can alter the behavior of adsorbents and the solubility of metals. Over time, these factors may compromise the efficacy of eggshell as an adsorbent.

### Limitations

1. Variability of organic matter:

- Different water sources: The organic content in water can vary significantly based on the source (e.g., industrial, agricultural runoff, or urban waste). This variability makes it challenging to predict the performance of ESW in different environmental conditions, requiring site-specific assessments.

2. Compounding contaminants:

- Complex mixtures: The presence of complex mixtures of contaminants (both organic and inorganic) can complicate the removal process. Interactions between different types of contaminants may lead to unforeseen challenges in the treatment process, which may require multi-faceted remediation strategies.

3. Limited longevity and regeneration of adsorbent:

- Saturation and regeneration: Over time, the organic load on eggshells may lead to saturation of adsorption sites, requiring regeneration processes that may not be environmentally friendly or feasible in low-resource settings. Determining effective regeneration protocols remains a challenge.

In brief, while the use of ESW as a bioremoval agent demonstrates substantial promise for addressing environmental contamination in water sources, the implications of organic content must be carefully considered. Challenges related to adsorption efficiency, nutrient release, and the variability of organic matter necessitate further research to optimize protocols and ensure consistent performance across diverse environmental conditions. Acknowledging these limitations will help refine strategies to leverage organic materials effectively in pollution remediation efforts.

### Novelty, future scope, and possible applications of eggshell waste in bioremediation

1. **Novelty** This study highlights the innovative use of eggshell waste (ESW), a readily available and environmentally friendly biowaste, as a natural adsorbent for the removal of potentially toxic elements and microbial contaminants from the raw water of the Nile River in Egypt. The novelty lies in leveraging an agricultural by-product that is traditionally considered waste and turning it into a sustainable solution for one of the pressing environmental issues: water contamination. Unlike conventional adsorbents, which may be costly and environmentally detrimental, ESW offers a sustainable alternative with high efficiency and low cost for removing contaminants such as lead, cadmium, and iron, as well as pathogenic microorganisms like *E. coli*. The study also provides detailed characterization of the adsorbent through techniques like Scanning Electron Microscopy (SEM) and Energy Dispersive X-ray analysis (EDX), significantly enhancing the understanding of its structural and compositional properties pre- and post-treatment, which has been relatively underexplored in previous studies using eggshell waste.
2. **Future scope** Given the promising results from the application of ESW as a bioremoval agent, several future avenues can be pursued to expand this research’s impact:

1. **Scaling up** Future research can explore large-scale applications of ESW in various water treatment facilities, including municipal wastewater treatment plants and agricultural runoff management systems. Conducting pilot projects will provide insights into the practicality and effectiveness of this bioremediation strategy in real-world scenarios.
2. **Diverse contaminant removal** Investigating the capacity of ESW for adsorbing a broader range of contaminants, including synthetic organic pollutants and emerging contaminants (like pharmaceuticals and personal care products), could enhance its applicability. Such studies could illuminate the mechanisms governing adsorption onto ESW for various chemical species.
3. **Synergistic effects** Future studies could explore the synergistic effects of combining ESW with other natural or engineered materials to further improve contaminant removal efficiency. This could include combining ESW with biochar or other biomaterials that also exhibit effective adsorption properties.
4. **Mechanistic studies** Detailed mechanistic studies focused on the adsorption-desorption dynamics and the interactions between contaminants and ESW could provide critical insights that inform modifications to improve performance further, such as surface modification or treatment of the eggshell before use.

3. **Possible applications** The findings from this study extend beyond water treatment, presenting ESW as a versatile material with several potential applications:

1. **Water treatment systems** ESW can be implemented in various water treatment systems as a green adsorbent material. This could range from small-scale household water purifiers to larger industrial applications, ensuring the removal of hazardous contaminants from drinking and irrigation water.
2. **Soil remediationl** Given its composition, ESW could also be utilized in soil remediation projects, serving as an amendment that not only enhances soil health but also adsorbs potential soil contaminants, mitigating the risks associated with agricultural runoff.
3. **Agricultural practices** Integrating ESW into agricultural practices could serve to both recycle waste and improve soil quality. Its use in composting or as a soil conditioner could enhance nutrient availability while simultaneously helping retain soil moisture.
4. **Waste management strategies** The application of ESW in waste management strategies can aid in the circular economy by transforming agricultural waste into a valuable resource for environmental remediation. This supports sustainability goals and reduces landfill dependency.

## Conclusion

This study explores the innovative use of eggshell waste (ESW) as a green adsorbent to remove toxic elements and microbial contaminants from the Nile River’s raw water, addressing water pollution while promoting sustainable waste management. The results show that ESW is highly effective, achieving 94.4% removal of lead, 64.7% for cadmium, and significant reductions in iron, in addition to completely eliminating *E. coli* within 24 h. This demonstrates ESW’s dual role as both an adsorbent and disinfectant. Comprehensive characterization using techniques like Scanning Electron Microscopy with Energy Dispersive X-ray analysis (SEM-EDX) offers insights into the structural changes and adsorption mechanisms during treatment. The research emphasizes ESW’s potential as a sustainable solution for water contamination and encourages further exploration of organic waste materials in environmental remediation. It advocates for optimization and scalability in water treatment applications to enhance water quality and public health outcomes.

## Data Availability

All data generated or analyzed during this study are included in this published article.

## Abbreviations

Ar: Arsenic
Cd: Cadmium
Cu: Copper
ESW: Eggshell waste
ESP: Eggshell waste powder
Fe: Iron
Hg: Mercury
h: Hour
min: Minutes
Mn: Manganese
Pb: Lead
SEM-EDX: Scanning Electron Microscopy with Energy Dispersive X-Ray Analysis
Zn: Zinc
